# Compliance and Attitudes towards the Gluten-Free Diet in Celiac Patients in Italy: What Has Changed after a Decade?

**DOI:** 10.3390/nu16152493

**Published:** 2024-07-31

**Authors:** Federica Fiori, Giulia Bravo, Susanna Neuhold, Giovanni Bartolone, Caterina Pilo, Maria Parpinel, Nicoletta Pellegrini

**Affiliations:** 1 Department of Medicine, University of Udine, 33100 Udine, Italy; federica.fiori@uniud.it (F.F.); brvgli@gmail.com (G.B.); maria.parpinel@uniud.it (M.P.); 2 Associazione Italiana Celiachia (AIC), 16124 Genoa, Italy; alimenti@celiachia.it (S.N.);; 3 Department of Agricultural, Food, Environmental and Animal Sciences, University of Udine, 33100 Udine, Italy

**Keywords:** behavior, adherence, health, limitations, social barriers, celiac disease

## Abstract

This study aims were (i) to describe Italian celiac patients who agreed to participate in the latest web survey and their attitudes toward the GF diet (compliance, perceived limitations, and worries) and (ii) to compare the answers given by the 2011 and 2022 responders. The self-administered questionnaire was distributed through the Italian Coeliac Association channels (link on social media, websites, and newsletters) to all of the celiac patients willing to participate in 2011 and 2022 (2427 and 3529 responders who answered the same questions, respectively). Descriptive analyses and the Pearson’s chi-squared test were performed. The responders were 1 to 84 years old and mainly female. The prevalence of adherent patients in 2022 was 91%, with the highest value (94%) in children (≤10 years old) and adolescents (15−17 years old). Overall, young adults were the most worried group. About a decade after the first survey, we observed a decreasing prevalence of transgression events (−5%) and (at least) occasional temptation (−17%), a decreasing prevalence of health-related and general worries, but an increasing prevalence of social life withdrawal. In conclusion, it is important to periodically monitor celiac patients’ compliance and attitudes towards the gluten-free diet. As also highlighted in international guidelines, a reorganization of the diagnosis/follow-up visits, including an expert dietary consultation, is needed.

## 1. Introduction

The estimated prevalence of celiac disease (CD) worldwide is about 0.7% [1], while, in Italy, it has risen from 0.23% in 2011 to 0.41% in 2021, affecting about 241.729 individuals [2].

The only treatment for CD, which is a chronic immune-mediated enteropathy triggered by gluten ingestion, is the gluten-free (GF) diet. An improvement in symptoms is generally observed in less than one month in patients with strict adherence to a GF diet [3]. Even if the availability of manufactured GF products increased in Italy in the last decades due an exponential growth of their market [4], it may be challenging to strictly adhere to the GF diet, since it requires the total avoidance of gluten-containing cereals such as barley, rye, wheat, and their derivates. Wheat in particular is widely used by the Italian population as an ingredient in traditional preparations (e.g., pasta, bread, and pizza) [5]. Moreover, gluten may be used as an additive in several manufactured foods and/or recipes and may be present in traces in various non-gluten-containing foods due to cross-contact. Cross-contact is also crucial in restaurant kitchens, where the cooks need to be properly trained on this topic to avoid it. There are also many psychological and social barriers to following a strict GF diet, such as the following: management of eating out during social events or when traveling, concerns about the cook’s knowledge of CD and cross-contact, and frustration at receiving adverse reactions from other people [6].

Since duodenal biopsies (i.e., the gold standard) are not a feasible method to use in routine follow-up [7], the most convenient tools to assess the adherence to the GF diet are structured simple questionnaires [8,9,10]. Even the most recent international guidelines recommend the use of standardized patient-reported adherence questionnaires as a reasonable method to assess patient adherence to the GF diet when an expert dietitian is not available to assess it by a direct dietetic evaluation, or when a comparison between populations is required [3]. However, several questionnaires are currently available in the literature, and the use of different ones implying different definitions of strict adherence makes it challenging to compare results [7,11,12]. With this limitation in mind, adherence to the GF diet varies worldwide between 42% and 91% in adult CD patients [12] and between 23% and 98% in children and adolescents [11]. In recent years, several studies have investigated adherence to the GF diet in Italian CD children and adolescents [7,13,14,15] or adults [16,17,18,19] using different questionnaires, reporting data of strict/excellent adherence ranging from 36% [15] to 95% [14]. However, the sample sizes of those studies were small (less than 200 subjects) and not representative of the Italian CD population. In addition, none of the cited authors conducted multiple surveys to understand how adherence to the GF diet changes over the years.

The present study is based on a questionnaire created by the Italian Coeliac Association—*Associazione Italiana Celiachia* (AIC). The AIC is a national association that provides advice, education, and support to celiac people to help them to follow a correct diet and improve their quality of life. As part of the AIC activities, data on CD patients have been collected through recurring nationwide web surveys [20]. In 2011, the year that this project began, approximately 14% of the sample reported violating the GF diet at least once in the previous month. Furthermore, about half of the sample reported thinking about transgression now and then [20].

The primary aim of the present study is to describe a large sample of Italian CD patients who agreed to participate in the 2022 web survey. The main focus of the study is to analyze differences in GF diet transgression behavior and on the worries and limitations experienced in following the GF diet by age group. The secondary aim is to compare the answers given by the 2011 [20] and 2022 CD responders on the same questions.

## 2. Materials and Methods

The survey was addressed to all responders willing to participate who self-reported that they had received an official diagnosis of CD. Patients with CD were reached through the AIC official online channels, and the questionnaire was available through a link disseminated via social media, national and regional websites, and AIC newsletters. It was compiled from 17 February 2022 to 22 July 2022. All data were anonymous and analyzed in aggregate form. This study was approved by the Institutional Review Board of the Department of Agricultural, Food, Environmental, and Animal Sciences of the University of Udine, Italy.

### 2.1. Questionnaire

The questionnaire was fully anonymized, and the estimated fill-in time was 10 to 15 min. The questionnaire was based on the former one [20], and it was updated to reduce the compiling time and to simplify data collection and analysis, e.g., reducing the number of open-answer questions in favor of multiple-choice questions with skip logic. The former questionnaire contained 69 main items. The present questionnaire had 50 main items. Some of them (N = 11) included multiple related questions displayed in a table, and some items (N = 6) included sub-questions with skip logic. Contrary to the previous version [20], all answers were mandatory. Most of the items were multiple choice with single (N= 34) or multiple answers (N = 8), while some items (N = 2 main items with multiple questions) included statements to rate using the Likert scale, from 1 (disagree) to 5 (agree). Only six main questions (years after the diagnosis, birth year, birth city and province, number of cohabitants, and years of AIC membership), and one sub-question (number of children) were open-answer questions.

The questionnaire could be divided into six sections by topic, as follows: (1) personal data (e.g., age, sex, marital status, education, household); (2) eating habits (e.g., location of meal consumption, type of meals/snacks); (3) perceived limitations due to GF diet (e.g., health, management of social occasions, everyday-life implications/renunciations); (4) perception of GF foods (e.g., food quality, main drivers in the choice of GF foods); (5) adherence to the GF diet (e.g., definition of transgression, transgression behavior, and its motivations); and (6) impact of AIC membership (e.g., membership status, quality of life, sources of information). In the present study, we analyzed 42 questions and sub-questions focused on sociodemographic and descriptive characteristics, health worries, practical constraints in adhering to the GF diet, sources of dietary information, and attitude toward transgression of the GF diet.

Since there were no exclusion criteria, the questionnaire could also be filled out by a minor. In this case, it was suggested that caregivers intervened as little as possible, leaving him or her free to answer each question independently and consciously, without conditioning.

### 2.2. Statistical Analysis

The main 2022 results were stratified by age group, as follows: group I includes children of 10 years old or younger; group II includes pre-adolescents from 11 to 14 years old; group III includes adolescents from 15 to 17 years old; group IV includes young adults from 18 to 39 years old; group V includes adults from 40 to 59 years old; and, finally, group VI includes adults older than 60 years (also referred to as the elderly). Descriptive analyses were performed for all variables. Data were reported as absolute and/or relative frequencies, calculated on the total number of responders or the total number of responders in each age group subsample. To evaluate how the difference between groups depended on chance, Pearson’s chi-squared test was performed. Analyses were conducted using the statistical software package SAS V.9.4 for Windows, and a two-tailed *p* value < 0.05 was considered statistically significant.

Finally, the distribution of the answers of the 2022 sample and of the 2011 subsample of CD patients (N = 2427), which included the same questions, was graphically compared.

## 3. Results

### 3.1. Descriptive Analysis of the Celiac Disease Sample of the Latest Survey

The 2022 survey involved 3529 CD responders, of which the majority were female and more than 99% were residents in Italy (Supplementary Figure S1).

The age varied between 1 and 84 years old, with 18–39 years old (age group IV; 1216 responders) and 40–59 years old (age group V; 1451 responders) being the most represented groups. Table 1 describes the characteristics of the six identified age groups and the total sample, excluding 5 responders who did not indicate their age (N = 3524). Most of the participants (83%) had a CD diagnosis from no less than 3 years before the questionnaire administration, and about 50% were diagnosed because of their severe clinical symptoms (Supplementary Figure S2). The other half were diagnosed because of mild symptoms, comorbidities, familiarity, infertility, or other reasons.

The majority of responders (84%) were members of the AIC, but only 49% of the sample declared that they keep themselves informed on CD and its symptoms. Younger responders (age groups I, II, and III, i.e., children and adolescents) reported more adherence to regular checks (91–94%) than adults (57–62%). Approximately 65% of the responders declared that they had received clear information about the GF diet at diagnosis (Supplementary Figure S3), while 5% of the sample had not received any information at all. Their main sources of information about the GF diet were AIC official channels, followed by non-AIC websites, and specialized magazines (Supplementary Figure S4). On the other hand, 43% and 71% of the sample declared that doctors or dietitians, respectively, were never a source of information on the GF diet during follow-up visits.

Looking at the adult population (Table 1), most of the sample had a high school diploma or higher degree, and 52% (age group IV), 71% (age group V), and 45% (age group VI) had a full-time occupation. Most of the young adults were single (64%) and had no children (82%), while the elderly were mainly married and had children. Regarding cohabitation, 47% of young adults still lived with their parents, 66% of adults (group V) lived with children alone or with other family members, and 46% of the elderly (group VI) lived with their partner only.

As shown in Table 2A, we found that a considerable number of CD patients reported believing that the GF diet limits their food choices (44% in the total sample; 52% in group III, i.e., adolescents). About two-thirds of the total sample declared that their food choices are at least occasionally determined by worries about their health, and more than half of the sample felt that their mood sometimes sways their food choices.

Only 23% of the responders stated that they never had to restrain their food consumption in the previous year because of the GF diet. In the two adult groups (IV and V), which had a comparable sample size, we found a different distribution in the answers, with more young adults declaring to frequently limit their food consumption than the group V adults. Similarly, we noted that the young adults were more frequently concerned when thinking about food and when choosing what to eat, and more frequently confused when entering a grocery shop than older adults. In addition, we observed that the frequency of agreeing to spend a lot of time thinking about what and where to eat decreased with age (38%, 22%, and 17% of young adults, adults, and the elderly, respectively). In detail, regarding where to eat, about half of the responders confirmed that they eat at home because they feel safer there, and more than one-third of the responders stated that is difficult to find restaurants that offer GF food. More young adults (10%) than adults (6%) declared that they frequently or always eat in restaurants without asking for information.

Regarding dietary habits, most of the responders declared that they do not follow any additional diet, while 24% of the total sample agreed that they would have different dietary habits if they were not affected by CD. However, only 9% affirmed that CD stops them from following another diet, including 11% among the young adults vs. 8% among the group V adults.

Along with dietary restrictions, some CD patients also suffered from restrictions in social life and at work (Table 2B). About two-thirds of the sample had declined an invitation at least once in the past year because they feared eating unsafe food, and about one-third declared that they usually avoid social situations where they might not be able to control the served food. More than one quarter of the sample declared that CD makes their interpersonal relations challenging, and that they currently go out with friends less frequently than they did before their diagnosis. In this regard, adolescents and young adults were more likely to disagree than children and adults, respectively, in going out less frequently than they did before their diagnosis. Finally, 12% (group IV) and 8% (group V) of young adults and adults believed that their diet has affected their chosen occupation.

When asked to define transgression of the GF diet, about half of the sample selected each of the three given definitions (Supplementary Figure S5). Only 59.0% of the sample selected the more general definition (i.e., transgression is eating foods that certainly contain gluten). The most chosen definition (63.5%) was that transgression is eating food that is known to be at risk of contamination.

Table 3 shows that 42% of the sample thought about transgressing the GF diet at least occasionally. We found a trend of decreasing temptation with age. Considering the two most represented groups, more young adults (group IV) than adults (group V) reported being tempted to violate the GF diet. When asked about their behavior in the previous month, 91% of the total sample said they had not violated the GF diet, with the highest percentages being found among children (group I) and adolescents (group III). Among the 9% that did transgress, about 6% (i.e., 62% of the transgressors) declared that they have transgressed once, and the rest declared that they have transgressed more than once. Almost 4% (i.e., 38% of the transgressors) declared they felt bad about it, 5% (i.e., 52% of the transgressors) felt that it was not a problem, and 1% (i.e., 10% of the transgressors) felt gratified. When asked about motivations, about half of the transgressors declared that it is difficult not to transgress (Supplementary Figure S6). Other popular answers were as follows: “occasional transgression does not affect my health”, “the GF diet is too harsh”, and “I am sick of the GF diet”. When transgressing, about half of the transgressors declared that they did not confess the fact to anyone or that they spoke about it to relatives only (Supplementary Figure S7). Furthermore, when stratifying the sample in those who were diagnosed because of severe symptoms vs. all other motivations (Supplementary Figure S2), we observed a statistically significant difference (*p* = 0.0121) in their transgression behavior (“In the last month, did you transgress the gluten-free diet?”), with more “yes” answers being found in the group without severe symptoms.

### 3.2. Comparison between 2022 and 2011

**Figure 1:**
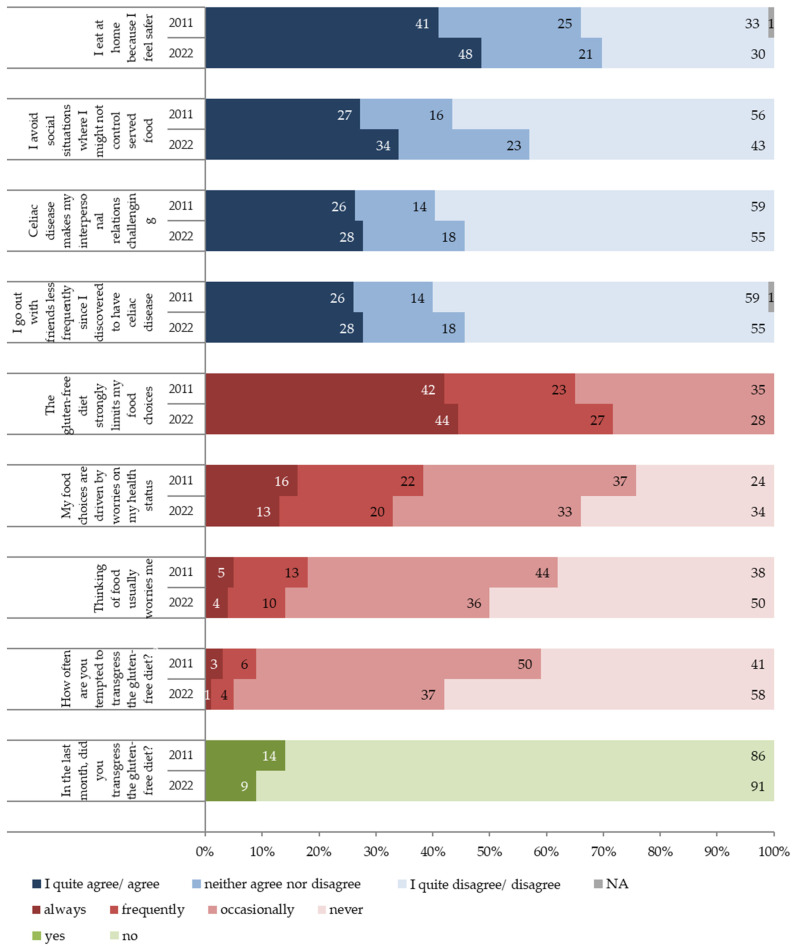
Attitude toward transgression of the gluten-free diet, health concerns, dietary limitations, and social life restrictions encountered in following the gluten-free diet in a follow-up perspective (2011–2022), expressed as the percentage of responders (2011, N = 2427; 2022, N = 3529).

Comparing the 2011 and 2022 answers of the two samples of CD patients, a higher percentage of people in 2022 than in 2011 felt that CD has an impact on their social life. Particularly, in 2022, more people agreed that they eat at home because they feel safer, and that they avoid social situations where they might not be able to control the served food (Figure 1).

Although responders in 2022 were generally more unsure (i.e., more neither agree nor disagree answers) than in 2011, a slightly higher percentage of patients in 2022 agreed that CD makes their interpersonal relations challenging and reported going out with friends less frequently. Moreover, fewer patients in 2022 than in 2011 declared that their diet never limits their food choices.

On the other hand, for health-related and general worries, a higher percentage of patients in 2022 than in 2011 declared that their worries about their health never drive their food choices and that thinking about food never worries them.

Fewer responders in 2022 than in 2011 (42% vs. 62%, respectively) said that they at least occasionally think about transgressing the GF diet. In both the 2011 and 2022 surveys, most responders affirmed that transgressing the GF diet involves eating foods while knowing that there is a risk of contamination or eating foods that certainly contain gluten (Supplementary Figure S5). Fewer people thought that GF diet transgression is eating without paying attention or eating foods without the GF claim. However, we noted a trend of rising consciousness on the definition of GF diet transgression from 2011 to 2022. Accordingly, more people in 2022 than in 2011 (91% and 86%, respectively) declared to have never transgressed the GF diet in the previous month (Figure 1).

## 4. Discussion

In this study, we described the behaviors, perceived limitations, and attitudes towards the GF diet in a large sample of CD patients of all age classes, providing a comprehensive overview of the situation in Italy. The results revealed a high prevalence of CD patients adhering to the GF diet, especially in children under ten years of age and adolescents. Young adults were the most worried group. Those that were more frequently tempted to transgress were children, preadolescents, adolescents, and young adults. When comparing our answers with those from the same questions administered to a similar sample of CD patients about a decade before, we found a trend towards a decreasing prevalence of transgression occasions and temptation to transgress, as well as a decreasing prevalence of health-related and general worries, but an increasing prevalence of withdrawal from social life.

Our sample was comparable to the Italian CD population [2] in terms of sex, age, and overall regional distribution. Compared to the national data [2], the northern regions were slightly more represented than the southern ones, and adults, aged 18 to 59 years old, (76% vs. 67%) were more represented than minors (14% vs. 22%) in our sample than in the national CD population. About 11% of our responders were older than 60 years. Accordingly, the national data show that only 11% of patients diagnosed with CD are older than 60 years [2]. Regarding sex distribution, female CD patients were slightly more represented among our responders than in the national CD population (75% vs. 70%).

Neither the definition of adherence to the GF diet nor the methodology for its assessment are univocally defined in the literature. In the present survey, we recorded the self-reported transgression frequency, which implies a subjective definition of transgression, mainly intended in our sample as eating foods while knowing that there is a risk of contamination both in manufactured foods and in social occasions that imply food consumption. As a result, adherence, intended as declaring to never indulge in transgressing the GF diet, ranged from 86% in both pre-adolescents and adults to 95% in children. Even if it appears that children and the elderly were more compliant than adults, we cannot find a clear trend among the age groups. Although the results are overall conflicting [21], the differences in adherence rates in specific age groups have been documented in the literature. In particular, lower adherence to the GF diet has been found in adolescence than in childhood [13,14]. Indeed, adolescence is known to be a critical period regarding transgression of the GF diet, since adolescents claim their independence from their caregivers [22,23]. On the contrary, in our survey, we found one of the highest adherence rates in group III (i.e., adolescents). The low prevalence of transgression found in this group may be explained by the fact that those who actively follow the AIC social media channels and decided to fill in the questionnaire may also be the more adherent ones. Regarding the adult population, in agreement with our data, a study on adult CD patients found an increasing adherence by age [19]. Young adults were also found to be more at risk of gluten consumption than other age groups in the 2011 survey [20].

Generally, despite different study designs and methodologies, comparable results were obtained in some Italian studies targeting children and adolescents [7,14] and adults [16], reporting percentages of strict/excellent adherence assessed by questionnaires exceeding 81%. However, we found a higher adherence rate than that reported in other studies, both in Italian children and adolescents [13,15] and in adults [17,18,19]. The high percentages found in our study may be partially due to a self-reporting bias [24].

Defining a gray zone of GF diet transgression, such as the percentage of patients who are occasionally to always (42%) tempted to violate the GF diet (“How often are you tempted to transgress the gluten-free diet?”), minus the percentage of patients (9%) who reported having violated the GF diet in the previous month (“In the last month, did you transgress the gluten-free diet?”), we found that 33% of our CD population was at risk of transgressing. This should be the target population for further interventions on raising awareness of the risks of even occasional transgression of the GF diet. Nevertheless, we are facing a positive trend, given that the gray zone decreased by 11 percentual points since 2011 [20]. This trend could be partially explained by the growing awareness of CD and the GF diet among the general population and food operators. This makes social acceptance and everyday life easier for people with CD. This has also contributed to the popularity of GF products, which are even perceived as healthier than their traditional counterparts by the general population, regardless of their nutritional composition [25]. The decreasing percentage of CD patients falling within the gray zone may also be explained by the growing presence of certified restaurants, cafeterias, and pizza houses offering GF foods in Italy [26,27]. However, despite the increasing GF food offering and the evident progress made in food technology, CD patients still seem to be dissatisfied with the taste, texture, price, and availability of GF products [28]. Accordingly, most of our responders declared that they had restricted their food consumption due to the lack of (tasty) GF alternatives at least once in the previous year (“In the last year, how often did you restrain your food consumption, e.g.,: I do not find/like the gluten-free alternative”).

When comparing 2011 to 2022 answers, improvements were observed in the answers on food and health concerns, but not in those on the social implications of CD. Indeed, more responders in 2022 than in 2011 said that they had reduced the frequency of social occasions and that they preferred to eat at home. This may also be a habit developed during the COVID-19 pandemic, when compliance with the GF diet improved in about one-third of CD patients in Italy, mainly due to the lower frequency of eating away from home and having more time to prepare food [29]. Eating away from home is known to be the domain where CD patients face most difficulties [6] and, therefore, is one of the main barriers to GF diet adherence [21]. Indeed, cross-contact in restaurants is a major problem for CD patients. Previous data show that chefs’ knowledge about CD was lower than that of the general population in the United Kingdom [30], but it has risen over the years [31].

Systematic reviews [11,12] found no association between adherence and the clinical symptoms of CD. However, the absence of severe symptoms when gluten is ingested may increase the struggle to follow the GF diet properly [32], as it may be misread as an indicator of irrelevant health implications. Indeed, the significant difference in transgression behavior observed when stratifying our sample based on their symptoms at the time of diagnosis indicates that the individuals with severe symptoms were less likely to transgress than the others. The lack of awareness of health implications was one of the main reasons for non-compliance with the GF diet in our population, as well as the difficulty in strictly following the diet. The difficulties experienced in following the GF diet may not only be due to practical limitations in everyday-life, but also to emotional and sociocultural barriers [12,21]. The correct management of social occasions by the CD population is an important determinant of their adherence to the GF diet [33]. Children and adolescents reported in a previous survey a lower adherence at certain social events than at home or at school, where they/their caregivers cannot directly control the served food (e.g., sleepovers and summer camps) [34]. Moreover, even if avoidance of social activities was not associated with better adherence [33], the struggle to follow the GF diet could be a deterrent to participating in those activities. Indeed, we found that most responders had declined an invitation for fear of eating unsafe food at least once in the previous year. In addition, our results suggest that adult CD patients feel less and less limited by their GF diet on social occasions as they become older. Indeed, the social burden is particularly evident in young adults, who feel more concerned than older adults about their diet, as confirmed by a previous study [16], and are more prone to eat in restaurants without asking for information about the served food. In this regard, a reluctance to manifest CD in public has been noticed, seeking social and professional acceptance [20,35]. Social fear and cultural factors are recognized to be important barriers to strict adherence to GF diets [21].

Other than being a source of social support, the associations of patients and their outputs/tools were found in the present study to be one of the preferred sources of information on the GF diet. Therefore, membership to a celiac association has been identified as a facilitator in improving dietary adherence [19,21]. However, the most significant facilitators reported were patient education and patient communication [21]. Alarmingly, in our sample, only half of the patients received clear information at the diagnosis on the consequences of not complying with the GF diet. It has been previously shown that CD patients who receive individual instructions on how to comply with the GF diet from healthcare providers [36] and those who attend regular dietetic follow-up visits [12] are less likely to transgress. Conversely, despite more than half of our sample adhering to regular checks, a considerable percentage of patients did not select the dietitian of the follow-up visit as a source of dietetic information. This may be due to the limited involvement of nutritional specialists in follow-ups, as found in a previous study in Greece, where contact with dietitians was revealed to be limited to the diagnosis [6]. In Italy, a recent study on a small sample of pediatric CD patients reported that only about 20% of the patients received dietary counselling from a trained dietitian at the diagnosis [7]. The same study highlighted the critical role that trained dietitians play during follow-up visits, as a consultation with a dietitian at follow-ups was associated with a stricter adherence to the GF diet [7]. The recently published evidence-based guidelines for the best practices in the monitoring of established CD in adult patients [3] confirm the importance of referral to expert dietitians or nutritionists since diagnosis to assess the nutritional, psychosocial, and anthropometric parameters and to ensure comprehensive dietary education, and during follow-up visits to review the parameters and evaluate patient adherence to the GF diet.

The main limitations of the present study are the use of a questionnaire, which was not formally validated, but that was repeated after a decade in a similar sample of CD patients [20], and the use of a self-administered questionnaire to assess the adherence to the GF diet (self-reporting bias). Other limitations are the unstructured definition of transgression, which was requested from the responders to obtain useful information on their awareness of the GF diet; the enrolment of the sample through the Italian Coeliac Association channels (84% of the responders were members, while the percentage of members among the Italian celiac population in 2022 was around 15%, according to unpublished AIC data), which may have possibly selected the most attentive CD patients (selection bias); the use of multiple-choice questions with the possibility of selecting more than one answer, which made the data analysis and interpretation difficult; and, finally, the unavailability of raw data from the 2011 survey, which prevented us from performing an additional statistical analysis comparing the answers to the 2011 and 2022 identical questions.

However, the present study has the merits of having analyzed a large sample that is comparable to the Italian CD population of all age classes, including the elderly, and having repeated the survey after about a decade, monitoring the changes in the attitudes towards the GF diet over the years.

## 5. Conclusions

The results of the present study from a large sample of the Italian CD population revealed a high prevalence of adherent CD patients and identified young adults as the group that might benefit the most from future tailored interventions. When comparing the 2011 and 2022 answers, we observed a trend towards a decrease in transgressions and in the temptation to transgress. Interestingly, despite the lower prevalence of transgression events declared and the lower prevalence of worries experienced by CD patients, the management of social occasions still appears to remain an important burden, leading to a reduction in social life activities.

Alarmingly we also noted that most CD patients did not rely on dietitians/nutritionists to gain information on their diet, in favor of online sources (mainly managed by the AIC). This highlights the need to reorganize the follow-up visits, moving toward international guidelines that recommend expert consultation both at the diagnosis and during follow-up visits to monitor adherence to the GF diet.

Monitoring adherence and attitudes towards the GF diet in the Italian CD population through a systematic and recurring survey plan (e.g., on a 5-year basis) could be extremely useful to focus resources where they are most needed. In addition, the future repetition of the present survey could assess whether the implementation of the latest guidelines is having the desired effect in improving adherence and reducing the GF diet burden. However, further work should be carried out to assess the adherence and attitudes towards the GF diet in a larger and more representative sample of adolescents. In this age group in particular, there is the need to minimize the selection bias, which has possibly influenced the results of the present and the previous literature studies by studying only the most attentive CD patients.

## Figures and Tables

**Table 1:** Characteristics of the responders by age group, expressed as number of respondents and percentage (N = 3524).

	Children and Adolescents			Adults		The Elderly	Total Sample
	I (≤10)	II (11–14)	III (15–17)	IV (18–39)	V (40–59)	VI (≥60)	
	N = 173	N = 163	N = 141	N = 1216	N = 1451	N = 380	N = 3524
	N (%)						
Sex							
Female	120 (69)	103 (63)	86 (61)	963 (79)	1091 (75)	273 (72)	2636 (75)
Male	52 (30)	57 (35)	54 (38)	243 (20)	352 (24)	104 (27)	862 (24)
I prefer not to answer	1 (1)	3 (2)	1 (1)	10 (1)	8 (1)	3 (1)	26 (1)
Years after diagnosis							
Less than 3 years	85 (49)	43(26)	29 (21)	205 (17)	178 (12)	32 (8)	572 (16)
3–10 years	85 (49)	109 (67)	73 (52)	405 (33)	451 (31)	103 (27)	1226 (35)
More than 10 years	0 (0)	8 (5)	38 (27)	601 (49)	817 (56)	245 (64)	1709 (48)
ND	3 (2)	3 (2)	1 (1)	5 (0)	5 (0)	0 (0)	17 (0)
Are you a member of the Italian Coeliac Association?							
Yes	136 (79)	125 (77)	106 (75)	992 (82)	1243(86)	347 (91)	2949 (84)
A relative of mine is a member	25 (14)	23 (14)	17 (12)	47 (4)	33 (2)	9 (29	154 (4)
No	12 (7)	15 (9)	18 (13)	177 (15)	175 (12)	24 (6)	421 (12)
Do you keep yourself informed on celiac disease?							
Frequently	104 (60)	93 (57)	83 (59)	539 (44)	721 (50)	200 (53)	1740 (49)
Occasionally	56 (32)	50 (31)	38 (27)	490 (40)	582 (40)	151 (40)	1367 (39)
Rarely	10 (6)	14 (9)	13 (9)	143 (12)	127 (9)	25 (7)	332 (9)
Never	3 (2)	6 (4)	7 (5)	44 (4)	21 (1)	4 (1)	85 (2)
Do you comply with regular checks?							
Yes, regularly	163 (94)	150 (92)	128 (91)	751 (62)	827 (57)	215 (57)	2234 (63)
Yes, occasionally	8 (5)	13 (8)	8 (6)	278 (23)	356 (25)	96 (25)	759 (22)
No	2 (1)	0 (0)	5 (4)	187 (15)	268 (18)	69 (18)	531 (15)
Level of education							
Primary school diploma/none	173 (100)	156 (96)	39 (28)	2 (0)	3 (0)	3 (1)	376 (11)
Secondary school diploma	0 (0)	7 (4)	99 (70)	94 (8)	93 (6)	34 (9)	327 (9)
High school diploma or equivalent	0 (0)	0 (0)	3 (2)	481 (40)	698 (48)	219 (58)	1401 (40)
University degree or higher	0 (0)	0 (0)	0 (0)	639 (53)	656 (45)	124 (33)	1419 (40)
ND	0 (0)	0 (0)	0 (0)	0 (0)	1 (0)	0 (0)	1 (0)
Main occupation							
Student/working student	173 (100)	163 (100)	141 (100)	333 (27)	5 (0)	0 (0)	815 (23)
Unemployed/searching for first job/occasional job	0 (0)	0 (0)	0 (80)	100 (8)	82 (6)	19 (5)	201 (6)
Housewife/househusband	0 (0)	0 (0)	0 (0)	25 (2)	100 (7)	157 (41)	282 (8)
Part-time employment	0 (0)	0 (0)	0 (0)	123 (10)	234 (16)	33 (9)	390 (11)
Full-time employment	0 (0)	0 (0)	0 (0)	635 (52)	1029 (71)	171 (45)	1835 (52)
ND	0 (0)	0 (0)	0 (0)	0 (0)	1 (0)	0 (0)	1 (0)
Marital status							
Single	173 (100)	163 (100)	141 (100)	775 (64)	228 (16)	32 (8)	1512 (43)
Life partner	0 (0)	0 (0)	0 (0)	195 (16)	150 (10)	15 (4)	360 (10)
Married	0 (0)	0 (0)	0 (0)	235 (19)	972 (67)	285 (75)	1492 (42)
Divorced/separated/widowed	0 (0)	0 (0)	0 (0)	11 (1)	101 (7)	48 (13)	160 (5)
Do you have any children?							
Yes	0 (0)	0 (0)	0 (0)	219 (18)	997 (69)	300 (79)	1516 (43)
No	173 (100)	163 (100)	141 (100)	997 (82)	454 (31)	80 (21)	2008 (57)
Who do you live with?							
Alone	0 (0)	0 (0)	0 (0)	100 (8)	126 (9)	57 (15)	283 (8)
With parents/parents and partner	169 (98)	161 (99)	139 (99)	568 (47)	77 (5)	3 (1)	1117 (32)
With partner/spouse only	0 (0)	0 (0)	0 (0)	261 (21)	261 (18)	173 (46)	695 (20)
With children/partner and children/partner, children, and children’s family	0 (0)	0 (0)	0 (0)	220 (18)	952 (66)	133 (35)	1305 (37)
With other relatives	4 (2)	2 (1)	2 (1)	18 (1)	10 (1)	7 (2)	43 (1)
With other cohabitants	0 (0)	0 (0)	0 (0)	49 (4)	25 (2)	7 (2)	81 (2)
Net monthly household income *							
EUR >2000	72 (42)	73 (45)	69 (49)	532 (44)	755 (52)	202 (53)	1703 (48)
EUR 1000–2000	41 (24)	31 (19)	25 (18)	479 (39)	486 (33)	139 (37)	1201 (34)
EUR <1000	6 (3)	6 (4)	5 (4)	126 (10)	140 (10)	25 (7)	308 (9)
None	54 (31)	53 (33)	42 (30)	79 (6)	69 (5)	14 (4)	311 (9)
ND	0 (0)	0 (0)	0 (0)	0 (0)	1 (0)	0 (0)	1 (0)
Are there other celiac people in your family?							
Yes	33 (19)	38 (23)	33 (23)	311 (26)	494 (34)	109 (29)	1018 (29)
No	140 (81)	125 (77)	108 (77)	905 (74)	957 (66)	271 (71)	2506 (71)

Abbreviation: ND, not declared. Notes: Data are presented as number of responders. The parentheses show the column percentage. * Given the high prevalence of no income declared in children and adolescents (groups I, II, and III), this question might have been misunderstood to be in reference to the CD patient and not to the household.

**Table 2:** Attitude towards dietary limitations, health worries (**A**), social, and occupational life restrictions encountered in following the gluten-free diet (**B**) by age group, expressed as number of respondents and percentage (N = 3524).

A.	Children and Adolescents			Adults		The Elderly	Total Sample
	I (≤10)	II (11–14)	III (15–17)	IV (18–39)	V (40–59)	VI (≥60)	
	N = 173	N = 163	N = 141	N = 1216	N = 1451	N = 380	N = 3524
	N (%)						
The gluten-free diet strongly limits my food choices							
I disagree/quite disagree	45 (26)	45 (28)	32 (23)	330 (27)	431 (30)	117 (31)	1000 (28)
Neither agree nor disagree	47 (27)	45 (28)	35 (25)	352 (29)	361 (25)	120 (32)	960 (27)
I quite agree/agree	41 (47)	73 (45)	74 (52)	534 (44)	659 (45)	143 (38)	1564 (44)
My food choices are driven by worries about my health status							
Never	*44 (25)*	*68 (42)*	*47 (33)*	403 (33)	500 (34)	137 (36)	1199 (34)
Occasionally	*63 (36)*	*51 (31)*	*44 (31)*	391 (32)	494 (34)	132 (35)	1175 (33)
Frequently	*41 (24)*	*26 816)*	*31 (22)*	267 (22)	267 (18)	67 (18)	699 (20)
Always	*25 (14)*	*18 (11)*	*19 (14)*	155 (13)	190 (13)	44 (11)	451 (13)
I think my mood sways my food choices							
Never	75 (43) **	78 (48) **	76 (54) **	487 (40)	577 (40)	187 (49)	1480 (42)
Occasionally	68 (39) **	59 (36) **	32 (23) **	345 (28)	444 (31)	119 (31)	1067 (30)
Frequently	21 (12) **	19 (12) **	21 (15) **	253 (21)	287 (20)	52 (14)	653 (18)
Always	9 (5) **	7 (4) **	12 (8) **	131 (11)	147 (10)	22 (6)	324 (9)
In the last year, how often did you restrain your food consumption (e.g.,: I do not find/like the gluten-free alternative)							
Never	44 (25)	37 (23)	40 (28)	264 (22) *	322 (22) *	104 (27)	811 (23)
Occasionally	60 (35)	81 (50)	63 (45)	463 (38) *	627 (43) *	161 (42)	1455 (41)
Frequently	53 (31)	30 (18)	30 (21)	406 (33) *	381 (26) *	74 (20)	974 (28)
Always	16 (9)	15 (9)	8 (6)	83 (7) *	121 (8) *	41 (11)	284 (8)
Thinking of food usually worries me							
Never	*66 (38)*	*80 (49)*	*58 (41)*	540 (44) *	789 (54) *	230 (60)	1763 (50)
Occasionally	*74 (43)*	*61 (37)*	*63 (44)*	449 (37) *	514 (35) *	121 (32)	1282 (36)
Frequently	*28 (16)*	*17 (10)*	*13 (9)*	164 (14) *	109 (8) *	24 (6)	355 (10)
Always	*5 (3)*	*5 (3)*	*7 (5)*	63 (5) *	39 (3) *	5 (1)	124 (4)
Having to pay attention to what to eat is a problem that worries me for more than an hour a day							
Never	*69 (40)*	*97 (60)*	*82 (58)*	684 (56) *	902 (62) *	268 (70)	2102 (60)
Occasionally	*76 (44)*	*39 (24)*	*42 (30)*	327 (27) *	365 (25) *	76 (20)	925 (26)
Frequently	*23 (13)*	*18 (11)*	*13 (9)*	134 (11) *	127 (9) *	25 (7)	340 (10)
Always	*5 (3)*	*9 (6)*	*4 (3)*	71 (6) *	57 (4) *	11 (3)	157 (4)
When I go to a grocery shop, I am confused							
Never	*84 (51)*	*106 (65)*	*91 (64)*	810 (67) *	1062 (73) *	312 (82)	2470 (70)
Occasionally	*77 (44)*	*45 (28)*	*42 (30)*	328 (27) *	333 (23) *	60 (16)	885 (25)
Frequently	*7 (4)*	*7 (4)*	*8 (6)*	63 (5) *	46 (3) *	7 (2)	138 (4)
Always	*0 (0)*	*5 (3)*	*0 (0)*	15 (1) *	10 (1) *	1 (0)	31 (1)
I spend a great deal of time thinking about where and what to eat							
I disagree/quite disagree	*75 (43)*	*86 (53)*	*82 (58)*	510 (42) *	852 (59) *	269 (71)	1874 (53)
Neither agree nor disagree	*42 (24)*	*36 (21)*	*29 (21)*	243 (20) *	284 (20) *	46 (12)	680 (19)
I quite agree/agree	*56 (32)*	*41(25)*	*30 (21)*	463 (38) *	315 (22) *	65 (17)	970 (28)
I eat at home because I feel safer							
I disagree/quite disagree	*32 (18)*	*49 (30)*	*48 (34)*	357 (29)	453 (31)	128 (34)	1067 (30)
Neither agree nor disagree	*39 (22)*	*30 (18)*	*31 (22)*	371 (22)	321 (22)	64 (17)	756 (21)
I quite agree/agree	*102 (59)*	*84 (51)*	*62 (44)*	588 (48)	677 (47)	188 (50)	1701 (48)
It is difficult to find restaurants that offer gluten-free food							
I disagree/quite disagree	*37 (21)*	*40 (24)*	*44 (31)*	390 (32) *	469 (32) *	128 (34)	1108 (31)
Neither agree nor disagree	*43 (25)*	*40 (24)*	*40 (28)*	358 (29) *	449 (31) *	130 (34)	1060 (30)
I quite agree/agree	*93 (54)*	*83 (51)*	*57 (40)*	468 (38) *	533 (37) *	123 (32)	1356 (38)
In the last year, how often did you eat in restaurants/pizzerias without asking for information on the served food?							
Never	*147 (85)*	*128 (78)*	*117 (83)*	885 (73) *	1107 (76) *	324 (85)	2708 (77)
Occasionally	*15 (9)*	*23 (14)*	*17 (12)*	211 (17) *	246 (17) *	41 (11)	553 (16)
Frequently	*8 (5)*	*5 (3)*	*7 (5)*	74 (6) *	62 (4) *	7 (2)	163 (5)
Always	*3 (2)*	*7 (4)*	*0 (0)*	46 (4) *	36 (2) *	8 (2)	100 (3)
Did you follow a specific diet other than the gluten-free diet?							
Yes	*13 (8)*	*5 (3)*	*11 (8)*	208 (17)	272 (19)	88 (23)	597 (17)
No	*160 (93)*	*158 (97)*	*130 (92)*	1008 (83)	1179 (81)	292 (77)	2927 (83)
If you were not a celiac patient, would you have the same food habits?							
Yes	*119 (69)*	*119 (73)*	*103 (73)*	933 (77)	1098 (76)	304 (80)	2676 (76)
No	*54 (31)*	*44 (27)*	*38 (27)*	283 (23)	353 (24)	76 (20)	848 (24)
Celiac disease stops me from following another diet (for example, vegan diet) that I followed in the past/I would have followed otherwise							
I disagree/quite disagree	*142 (82)*	*134 (82)*	*118 (84)*	980 (81) *	1246 (86) *	315 (83)	2935 (83)
Neither agree nor disagree	*22 (13)*	*14 (9)*	*12 (8)*	101 (8) *	89 (6) *	23 (6)	261 (7)
I quite agree/agree	*9 (5)*	*15 (9)*	*11 (8)*	135 (11) *	116 (8) *	42 (11)	328 (9)
**B.**	**Children and Adolescents**			**Adults**		**The Elderly**	**Total** **Sample**
	**I (≤10)**	**II (11–14)**	**III (15–17)**	**IV (18–39)**	**V (40–59)**	**VI (≥60)**	
	**N = 173**	**N = 163**	**N = 141**	**N = 1216**	**N = 1451**	**N = 380**	**N = 3524**
	**N (%)**						
In the last year, how often did you turn down an invitation for fear of eating unsafe food?							
Never	39 (23)	56 (34)	43 (31)	358 (29) *	484 (33) *	114 (30)	1094 (31)
Occasionally	84 (49)	58 (36)	63 (45)	495 (41) *	605 (42) *	153 (40)	1458 (41)
Frequently	44 (25)	39 (24)	26 (18)	304 (25) *	281 (19) *	86 (23)	780 (22)
Always	6 (4)	10 (6)	9 (6)	59 (5) *	81 (6) *	27 (7)	192 (5)
I avoid social situations (parties, happy hours, etc.) where I might not control served food							
I disagree/quite disagree	69 (40)	82 (50)	63 (45)	575 (47)	618 (43)	123 (32)	1530 (43)
Neither agree nor disagree	50 (29)	47 (29)	37 (26)	262 (22)	338 (23)	75 (20)	809 (23)
I quite agree/agree	54 (31)	34 (21)	41 (29)	379 (31)	495 (34)	182 (48)	1185 (34)
Celiac disease makes my interpersonal relations challenging							
I disagree/quite disagree	93 (54)	88 (54)	73 (52)	624 (51)	810 (56)	231 (61)	1919 (54)
Neither agree nor disagree	40 (23)	33 (20)	33 (23)	260 (21)	278 (19)	61 (16)	705 (20)
I quite agree/agree	40 (23)	42 (26)	35 (25)	332 (27)	363 (25)	88 (23)	900 (26)
I go out with friends less frequently since I discovered that I have celiac disease							
I disagree/quite disagree	78 (45) **	83 (51) **	88 (62) **	732 (60) *	747 (52) *	198 (52)	1926 (55)
Neither agree nor disagree	46 (27) **	42 (26) **	20 (14) **	210 (17) *	248 (17) *	61 (16)	627 (18)
I quite agree/agree	49 (28) **	38 (23) **	33 (23) **	274 (22) *	456 (31) *	121 (32)	971 (28)
My diet affects the occupation I have chosen/I want							
I disagree/quite disagree	*134 (78)*	*111 (68)*	*98 (70)*	945 (78) *	1192 (82) *	309 (81)	2789 (79)
Neither agree nor disagree	*26 (15)*	*32 (20)*	*19 (14)*	126 (10) *	135 (9) *	32 (8)	370 (10)
I quite agree/agree	*13 (8)*	*20 (12)*	*24 (17)*	145 (12) *	124 (8) *	39 (10)	365 (10)

Notes: Data are presented as number of responders. The parentheses show the column percentage. * Values are significantly different between adults (groups IV and V), according to Pearson’s chi-squared test (*p* < 0.05). ** Values are significantly different among children and adolescents (groups I, II, and III), according to Pearson’s chi-squared test (*p* < 0.05). The test was not performed for questions whose values are highlighted in italics in children and adolescents (groups I, II, and III).

**Table 3:** Attitude towards transgression of the GF diet by age group, expressed as number of respondents and percentage (N = 3524).

	Children and Adolescents			Adults		The Elderly	Total Sample
	I (≤10)	II (11–14)	III (15–17)	IV (18–39)	V (40–59)	VI (≥60)	
	N = 173	N = 163	N = 141	N = 1216	N = 1451	N = 380	N = 3524
	N (%)						
How often are you tempted to transgress the gluten-free diet?							
I never think about it	89 (51)	74 (45)	66 (47)	652 (54) *	897 (62) *	263 (69)	2041 (58)
Occasionally	74 (43)	77 (47)	67 (48)	489 (40) *	496 (34) *	108 (28)	1311 (37)
Frequently	8 (5)	12 (7)	8 (6)	62 (5) *	47 (3) *	6 (2)	143 (4)
Always	2 (1)	0 (0)	0 (0)	13 (1) *	11 (1) *	3 (1)	29 (1)
In the last month, did you transgress the gluten-free diet?							
Yes	11 (6)	14 (9)	8 (6)	132 (11)	136 (9)	25 (7)	326 (9)
No	162 (94)	149 (91)	133 (94)	1084 (89)	1315 (91)	355 (93)	3198 (91)
In the last month, how often did you transgress the gluten-free diet?							
Yes, once	8 (5)	9 (6)	6 (4)	76 (6)	85 (6)	18 (5)	202 (6)
Yes, sometimes	3 (2)	5 (3)	2 (1)	43 (4)	43 (3)	7 (2)	103 (3)
Yes, many times	0 (0)	0 (0)	0 (0)	13 (1)	8 (1)	0 (0)	21 (1)
I did not transgress the gluten-free diet in the last month	162 (94)	149 (91)	133 (94)	1084 (89)	1315 (91)	355 (93)	3198 (91)
When you transgressed, how did you feel?							
I felt bad because I felt guilty	5 (3)	5 (3)	2 (1)	53 (4)	52 (4)	7 (2)	124 (4)
It was not a problem	4 (2)	7 (4)	4 (3)	72 (6)	69 (5)	14 (4)	170 (5)
I felt good because I was gratified	2 (1)	2 (1)	2 (1)	7 (1)	15 (1)	4 (1)	32 (1)
I did not transgress the gluten-free diet in the last month	162 (94)	149 (91)	133 (94)	1084 (89)	1315 (91)	355 (93)	3198 (91)

Notes: Data are presented as number of responders. The parentheses show the column percentage. * Values are significantly different between adults (groups IV and V), according to Pearson’s chi-squared test (*p* < 0.05).

## Data Availability

The data presented in this study are available upon request from the corresponding author. Raw data are not publicly available due to the presence of information that could compromise the privacy of research participants.

## Supplementary Materials

The following supporting information can be downloaded at: https://www.mdpi.com/article/10.3390/nu16152493/s1, Figure S1: Residential region of the celiac disease responders (N = 3529); Figure S2: Reasons that led to the celiac disease diagnosis (N = 3529); Figure S3: Information received at the diagnosis (N = 3529). Multiple answers were allowed; Figure S4: Sources of information about the gluten-free diet (N = 3529). Multiple answers were allowed; Figure S5: Definition of “gluten-free transgression” chosen by the responders in 2011 (N = 2427) and 2022 (N = 3529). Multiple answers were allowed; Figure S6: Motivations for gluten-free diet transgression (N = 326). Multiple answers were allowed; Figure S7: The person to whom celiac disease patients confess their transgression (N = 326). Multiple answers were allowed.
