# Supplementary material for: Compliance and Attitudes towards the Gluten-Free Diet in Celiac Patients in Italy: What Has Changed after a Decade?
Source: Nutrients. 2024 Jul 31;16(15):2493. doi: 10.3390/nu16152493 (PMC11314567; doi:10.3390/nu16152493)
Supplement: Supplementary file 1 [file nutrients-16-02493-s001.zip › nutrients-3105497-supplementary.pdf]

SUPPLEMENTARY MATERIALS

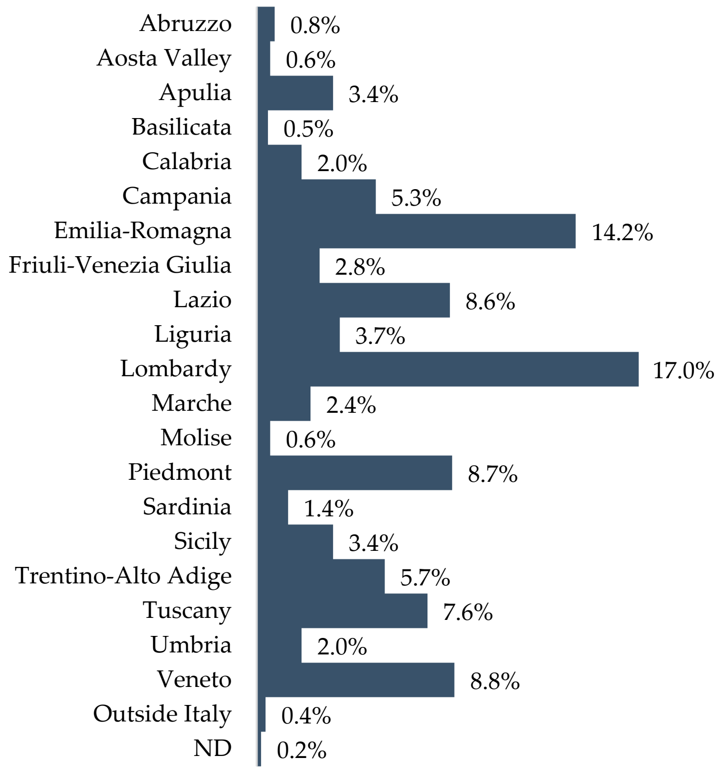

Supplementary Figure S1: Residential region of the celiac disease responders (N=3529).

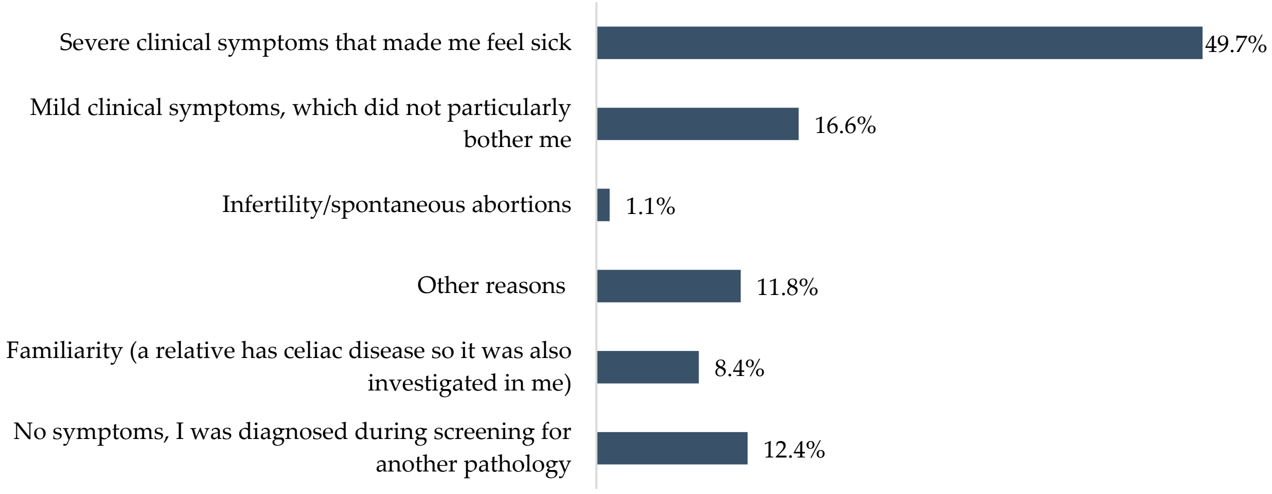

Supplementary Figure S2: Reasons that led to the celiac disease diagnosis (N=3529).

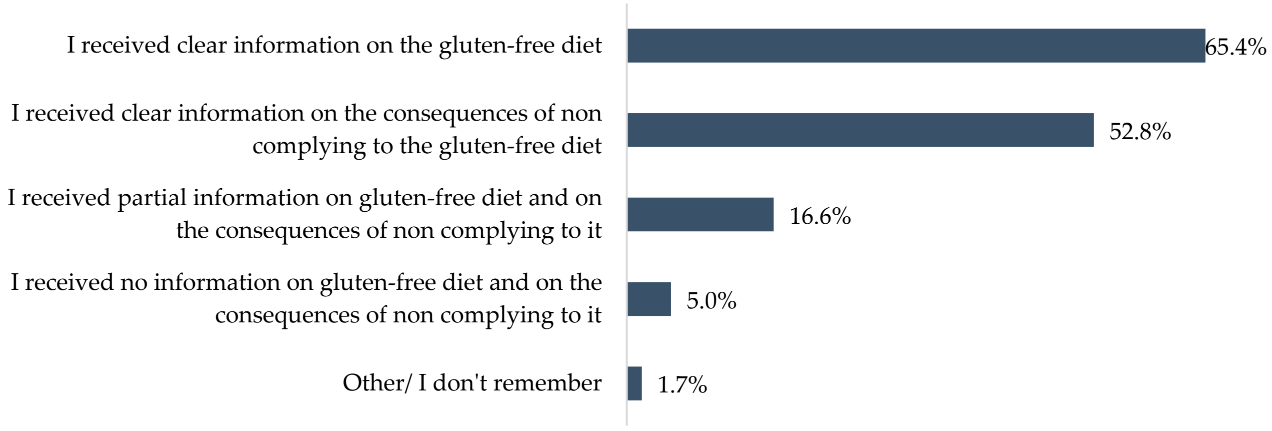

Supplementary Figure S3: Information received at the diagnosis (N=3529). Multiple answers were allowed. Abbreviations: AIC, Italian Celiac Association—*Associazione Italiana Celiachia*

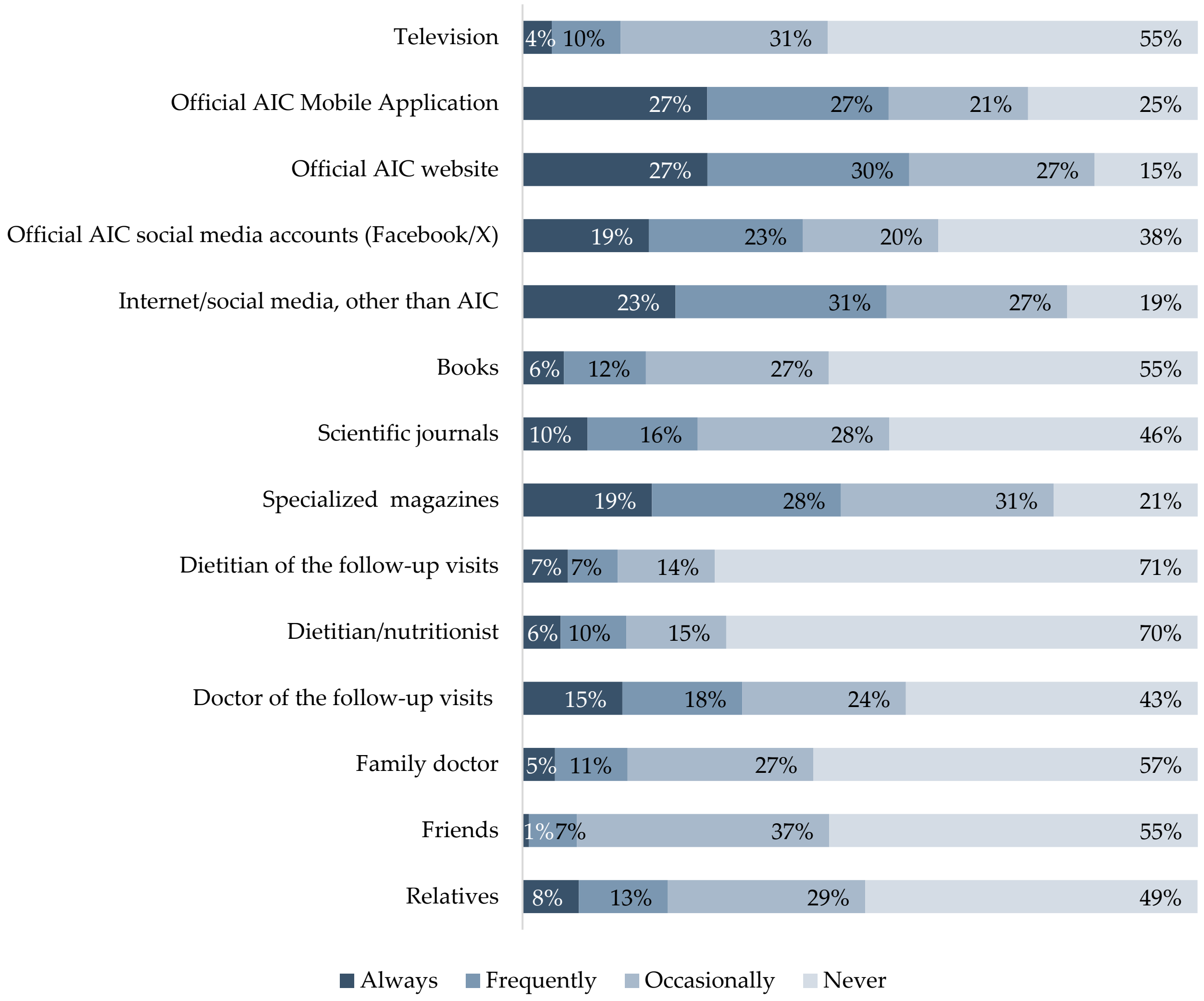

Supplementary Figure S4: Sources of information about the gluten-free diet (N=3529). Multiple answers were allowed.

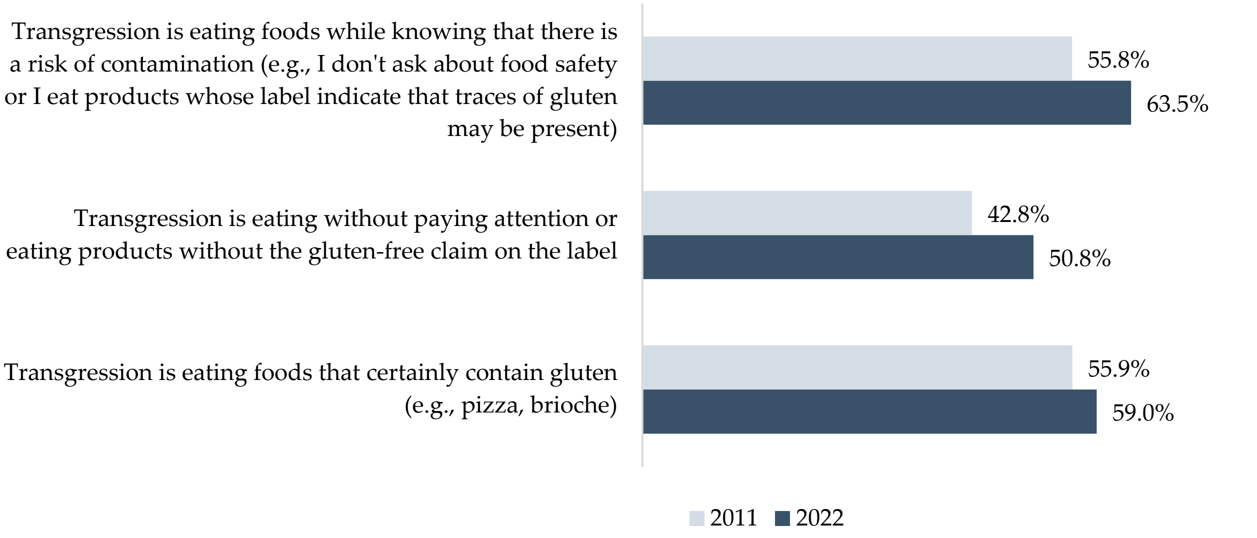

Supplementary Figure S5: Definition of "gluten-free diet transgression" chosen by the responders in 2011 (N=2427) and 2022 (N=3529). Multiple answers were allowed.

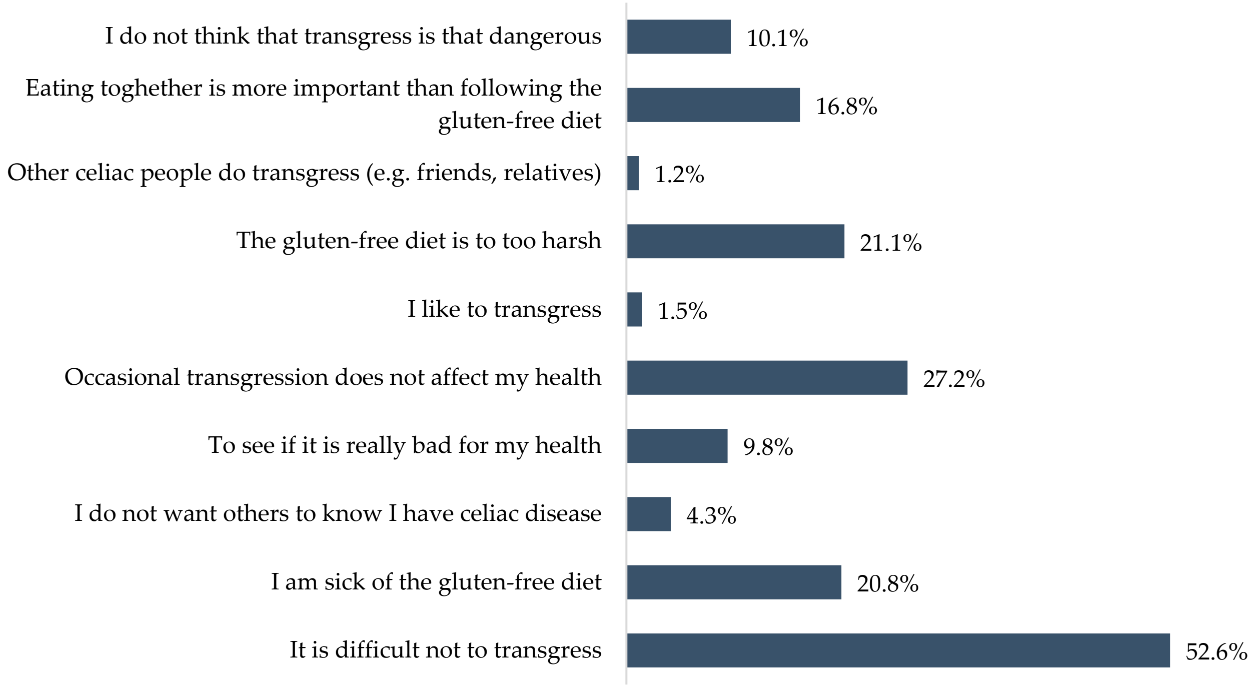

Supplementary Figure S6: Motivations for gluten-free diet transgression (N=326). Multiple answers were allowed.

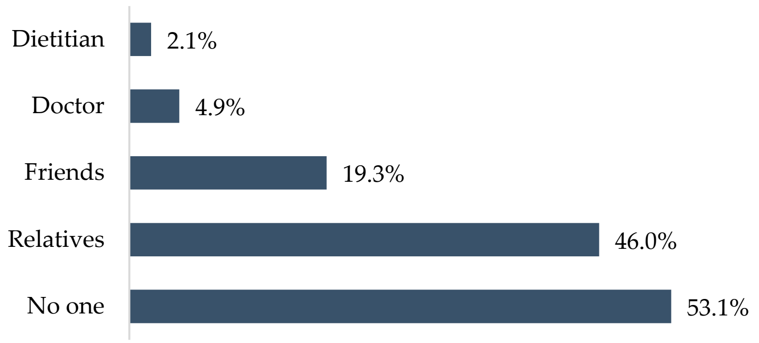

Supplementary Figure S7: The person to whom celiac disease patients confess their transgression (N=326). Multiple answers were allowed.
